# Management of Primary Spontaneous Pneumothorax in a Third-Level Pediatric Surgical Center: A Retrospective Study

**DOI:** 10.3389/fped.2022.945641

**Published:** 2022-06-27

**Authors:** Giovanna Spezzotto, Alessandro Boscarelli, Manuela Giangreco, Giulia Ceschiutti, Daniela Codrich, Maria-Grazia Scarpa, Marianna Iaquinto, Damiana Olenik, Edoardo Guida, Jürgen Schleef

**Affiliations:** ^1^Faculty of Medicine and Surgery, University of Trieste, Trieste, Italy; ^2^Department of Pediatric Surgery and Urology, Institute for Maternal and Child Health - IRCCS “Burlo Garofolo”, Trieste, Italy; ^3^Clinical Epidemiology and Public Health Research Unit, Institute for Maternal and Child Health - IRCCS “Burlo Garofolo”, Trieste, Italy; ^4^Surgical Department, Institute for Maternal and Child Health - IRCCS “Burlo Garofolo”, Trieste, Italy

**Keywords:** primary spontaneous pneumothorax, children, management, conservative treatment, surgery

## Abstract

**Introduction:**

The management of primary spontaneous pneumothorax (PSP) in pediatrics remains controversial. The aim of this study was to investigate the risk of recurrence after non-surgical treatment vs. surgery, the difference in the length of stay (LOS) between various treatment options, and the role of computed tomography (CT) in the management of PSP.

**Materials and Methods:**

We retrospectively reviewed patients admitted to our Pediatric Surgery Unit for an episode of PSP between June 2009 and July 2020. Medical records including clinical presentation at admission, diagnostics, treatments, complications, and LOS were collected.

**Results:**

Twenty-three patients (22 males and 1 female) were included in this study. Median age was 15.65 (range 9–18). Chest X-rays were performed in all patients and showed 5 small (22%) and 18 large (78%) PSP. Chest drain was used for large PSP (≥2 cm) if the patient was clinically unstable. Eleven patients (48%) were managed non-operatively with observation alone and a recurrence rate of 18%, chest drain was used in 11 patients with a recurrence rate of 36%, and surgery was deemed necessary as a first treatment choice in one case. Six patients (27%) had an episode of relapse after non-operative management or chest drain placement. Following surgery, a relapse occurred in 2 of the 6 patients. Chest drain insertion was associated with a longer LOS than observation alone (6.36 vs. 2.4 days), and surgery resulted in a longer LOS than other types of treatment (*P* = 0.001).

**Conclusion:**

According to our experience, small PSP or clinically stable larger PSP can be treated conservatively with observation alone. Operative management should be taken into consideration in children with large symptomatic PSP, persistent air leak, and/or relapse after chest drain insertion.

## Introduction

Primary spontaneous pneumothorax (PSP) is defined as a spontaneously occurring pneumothorax in the absence of prior lung disease or trauma. The incidence of PSP has been reported to be around 3.4/100,000 in childhood, with a male to female ratio of 4:1 (1). Tall, thin boys with a below average body mass index (BMI) are typically affected (2). While it is widely accepted that blebs, bullae, or emphysema-like changes play a large role, the exact etiology remains to be elucidated (3).

The role of computed tomography (CT) is controversial. Some studies have noted that the risk of recurrence is much higher in patients with bullae and blebs visible at CT than in those without (4), while other studies have not found this match. Additionally, according to some authors, finding bullae and blebs at CT scan is not an indication for surgery (5). In the pediatric population, the high dose of radiation to which patients are subjected during CT should be taken into consideration.

The management of PSP in children remains debated and based on evidence from adult patients. Several studies have been published concerning the treatment of pneumothorax in children. However, their limitations include being largely retrospective and following a small number of patients. Initial management for small PSP is usually conservative, with observation alone and/or oxygen supplementation. By contrast, large PSP is typically treated with chest drain insertion, with surgery reserved for those cases presenting air leak or failure of the aforementioned strategies. Traditional thoracotomy surgery has been associated with a reduction of relapse but increased morbidity, LOS, and mortality. With the introduction of video-assisted thoracoscopic surgery (VATS), episodes of relapse have been significantly reduced. Some studies support that VATS is a safe treatment and should be considered if the air leak persists or in case of relapse (6–9).

The purpose of this study was to examine the best treatment strategy in terms of LOS and risk of relapse, and to establish the role of CT in the management of PSP.

## Materials and Methods

We retrospectively reviewed patients <18 years of age admitted to our Pediatric Surgery Unit for PSP from June 2009 to July 2020. Data were extrapolated from the departmental database and clinical notes were reviewed. Patients with traumatic or iatrogenic pneumothorax and underlying lung disease (asthma, pneumonia, cystic fibrosis, malignancy, connective diseases) were excluded. Demographic data (age and sex), clinical presentation at admission, diagnostics (X-ray and CT), primary and follow-up treatments (conservative, surgery), complications, and length of hospitalization were examined. X-ray study was performed in all patients and PSP size was calculated based on BTS guidelines. A “small” pneumothorax was defined as having a visible rim <2 cm between the lung margin and the chest wall (at the level of the hilum), while a “large” pneumothorax showed a rim ≥2 cm (10). CT scan was performed after a single episode of recurrence or in those patients with a history of spontaneous pneumothorax. Small pneumothorax was treated with observation alone, large pneumothorax was treated with chest drain, and surgery was performed in patients with relapse. Outcomes considered were recurrence rate post observation alone and post drain insertion, length of hospitalization related to a specific type of treatment, secondary treatments, and timing of CT scan.

### Statistical Analysis

Fisher's exact test was used to analyze the relationship between categorial variables, and the Kruskal-Wallis test was used to analyze the relationship between continuous variables. A significance level of *P* < 0.05 was chosen.

## Results

Twenty-three patients, 22 males and 1 female, admitted to our Pediatric Surgery Unit with a first episode of PSP were included in this study. Mean age was 15.65 (range 9–18 years). One patient had a family history of pneumothorax, and one patient had a smoking habit. Twenty-one patients (92%) presented with a pneumothorax on the left side, one on the right side and one on both sides. Chest X-rays were performed in all patients and showed 5 small (22%) and 18 large (78%) PSP. None of the patients with small PSP relapsed. CT scan was performed in 9 cases (39%), 7 of them after a relapse (78%), and 6 of the 9 children (67%) presented apical blebs or bullae. All patients with blebs or bullae detected at CT scan had at least one episode of recurrence, but statistical analysis using Fisher's exact test showed this relationship was not significant (*P* = 0.08). Chest drain was used for large PSP (≥2 cm) if a patient was clinically unstable, presenting with dyspnea and significant hypoxia. Eleven patients (48%) were managed non-operatively with observation alone and a recurrence rate of 18%, chest drain was used in 11 patients with a recurrence rate of 36%, and surgery was deemed necessary as a first treatment choice in one case. Chest drain insertion was associated with a longer LOS than observation alone (6.36 vs. 2.4 days), and surgery resulted in a longer LOS than other types of treatment (*P* = 0.001). Six patients (27%) had an episode of relapse after non-operative management or chest drain placement in a median time of 12 months (range 1–24 months); 4 experienced an ipsilateral pneumothorax, one bilateral pneumothorax and one contralateral pneumothorax. Relapses were not related to the type of therapeutic strategy (*P* = 0.24). Thoracoscopic surgery was performed in 6 patients, in 5 cases after a relapse episode and in one case as a first therapeutic approach in a child with a history of recurrent pneumothorax. Indications for surgical intervention were recurrent ipsilateral pneumothorax (50%), contralateral pneumothorax (17%), and bilateral pneumothorax (33%). We performed a thoracoscopic procedure with a pulmonary wedge resection of the apical lung in 4 cases (67%), of which 2 were associated with mechanical pleurodesis and 2 with chemical pleurodesis. Two patients underwent apicectomy alone (33%). Following the surgical procedure, a relapse occurred in 2 of 6 patients (33%) with subsequent further relapses. In both cases, CT scan detected blebs or bullae, confirming that these lesions are involved in recurrence episodes. All variables analyzed are summarized in Tables 1, 2.

**Table 1:** Patient characteristics, management, and outcomes after recurrence.

**Characteristics**	**Results**
**Age, median (IQR)**
	16 (15–17)
**Sex**, ***n*** **(%)**
Female	1 (4)
Male	22 (96)
**Smokers**, ***n*** **(%)**
	1 (4)
**Familiarity**, ***n*** **(%)**
	1 (4)
**Side**, ***n*** **(%)**
Left	21 (92)
Right	1 (4)
Bilateral	1 (4)
**Size**, ***n*** **(%)**
Small (<2 cm)	5 (22)
Large (≥2 cm)	18 (78)
**CT scan**, ***n*** **(%)**
	9 (39)
**Bullae/blebs found at CT scan**, ***n*** **(%)**
	6 (67)
**Management**, ***n*** **(%)**
Observation alone	11 (48)
Chest drain	11 (48)
Surgery	1 (4)
**Relapse**, ***n*** **(%)**
After observation	2 (18)
After chest drain	4 (36)
After surgery	1 (100)
**Management after relapse**, ***n*** **(%)**
Observation alone	1 (14)
Chest drain	0 (0)
Surgery, *n* (%)	6 (86)

*IQR, interquartile range; n, number; CT, computed tomography*.

**Table 2:** Management-based results and CT-findings after recurrence.

**Variables**	**Management**			***P*-value**
	**Observation alone**	**Chest drain**	**Surgery**	
**Relapse**, ***n*** **(%)**
Yes	2 (18.2)	4 (36.4)	1 (100.0)	0.24^*^
No	9 (81.8)	7 (63.6)	0 (0.0)	
**Lenght of stay, median (IQR)**
	2 (2–3)	5 (4–8)	10 (10–10)	0.001^**^
**Variables**				***P*** **-value**
**Blebs/Bullae at CT scan**
**after a relapse**, ***n*** **(%)**
Yes	6 (100.0)			0.08^*^
No	0 (0.0)			

* *Fisher exact test*.

** *Kruskall Wallis test*.

*n, number; IQR, interquartile range; CT, computed tomography*.

## Discussion

PSP is rarer in the pediatric population than in adults. It generally occurs in tall, thin males aged 10–30 (11). In our study, the mean age was approximately 16 years. Unfortunately, we do not routinely perform genetic workup and/or evaluation in patients admitted to our pediatric surgery unit for PSP, therefore we have not investigated any possible association with relapse rate in our study. Smoking is implicated in the development of PSP, and the dangerous effect of tobacco has been recognized. Some authors highlighted that most adult patients (up to 75%) (12) with PSP were smokers, but in our study only one patient reported a smoking habit.

The etiology of PSP remains unclear. It is well accepted that PSP occurs from the rupture of blebs and bullae on the apex of the lung. The formation of these lesions is most likely multifactorial, including physical characteristics, anatomic abnormalities of the bronchial tree, genetic factors, and growth (13, 14). Fujino et al. (15) suggested the hypothesis that growth during adolescence rapidly increases the vertical dimension of the thorax, causing an increase in negative pressure at the apex of the lung that may lead to formation of subpleural blebs or fluid-filled cysts with consequent pneumothorax upon rupturing. These lesions are not usually visible on X-ray but can often be detected using CT.

Diagnosis of PSP is generally clinical and confirmed by X-ray. In our study, like many others, CT was not usually performed at admission (16–18). Some authors argue that finding blebs at CT, even if performed for other indications, is a strong indication for surgery (19). In contrast, most authors state that CT evidence of bullae does not necessarily predict outcomes, even if they agree that CT is an effective method to delineate the anatomical status of the bullae and bleb formation (20, 21). Another consideration before undertaking CT scanning is the large amount of ionizing radiation involved.

In our study the biggest bleb size was 19 mm, while the average size was 4–5 mm. In all patients undergoing CT evaluation after a relapse, blebs and/or bullae were highlighted. However, the statistical analysis did not detect significance between recurrent episodes and lung injures (*P* = 0.08). Since the results showed no correlation between the presence of these lesions and the possibility of recurrence, there is no evidence that performing a CT scan on admission predicts future relapses. This supports our idea of reserving CT for cases of persistent air leak and relapse due to the high radiation dose involved in this imaging technique. Nonetheless, CT can assist in choosing the best therapeutic approach in selected cases.

The largest problem in PSP management is the lack of standardized guidelines. Consequently, PSP treatment is based on the experience of the individual surgery unit and evidence from the adult population. The main issues are choosing between observation alone with or without oxygen administration or chest drain insertion, and the correct timing for surgery. To date, the initial treatment approach is decided according to the size and symptoms at admission. The current management for small PSP is based on observation with oxygen administration in the majority of cases. By contrast, the presence of a large (≥2 cm) pneumothorax is the main indication for insertion of a chest drain. The therapeutic strategy used in our center for small PSP and clinically stable large PSP was simple observation. Despite this, the length of hospitalization of our patients was similar compared to patients treated with oxygen administration in other studies (1, 6, 9, 16). These patients generally underwent chest X-ray within 3 days of hospitalization to check if the pneumothorax had been reduced in size and to determine the possibly of discharge. In case of failure of conservative treatment (in terms of increase in size and/or exacerbation of symptoms such as dyspnea or hypoxia), we opted for the insertion of a chest drain. The LOS was longer in patients treated with chest drain insertion compared to those who were only observed (6.36 vs. 2.4 days), and this appears to be in keeping with other studies (8, 16). The longest LOS was related to surgery (10 days). Our statistical analysis showed that the LOS is linked to the therapeutic strategy (*P* = 0.001), confirming that it would be advisable to initially opt for a non-invasive treatment of observation alone in those patients with a small PSP and/or clinically stable to reduce the length of hospitalization.

**Figure 1:**
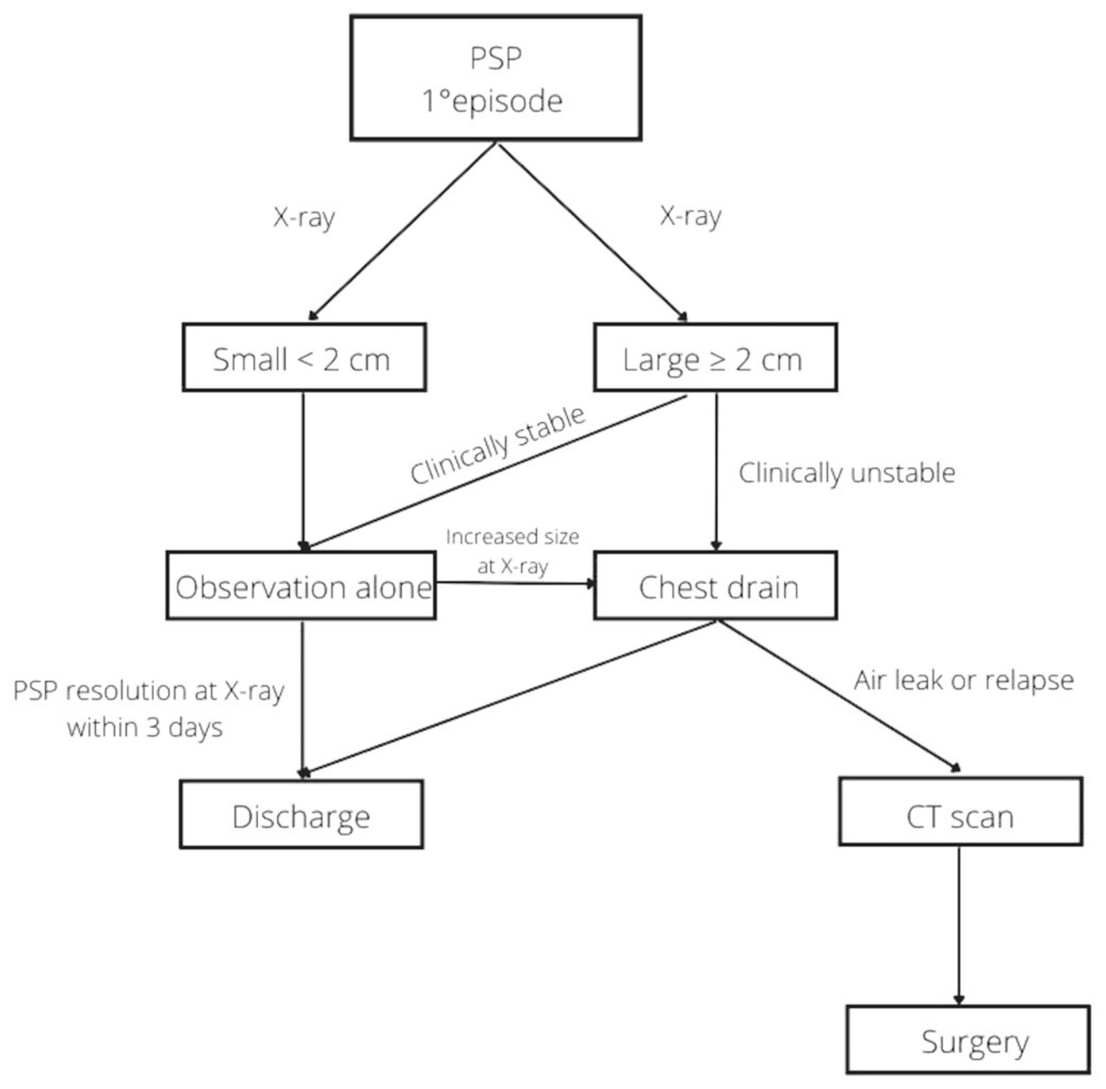
Flow chart for management of pediatric patients with primary spontaneous pneumothorax at our institution.

Non-surgical management is still associated with a high failure rate. In our study, 27% of patients had an episode of relapse, but the incidence was even higher in other studies (1, 16). The statistical analysis showed that the possibility of relapse was not associated with the initial treatment chosen (*P* = 0.24). This confirms that patients with small and/or clinically stable larger PSP may be treated with observation alone, reserving more invasive treatments for any clinical exacerbation, persistent air leak and/or relapse. VATS was first reported by Rodgers in 1986 for the treatment of secondary pneumothorax in children with cystic fibrosis (22). This surgical advancement has completely changed the approach to surgery for the treatment of PSP. Prior to VATS, traditional surgery involved high morbidity and mortality. However, VATS is considered a safe method with significantly reduced morbidity and mortality (1, 16, 23, 24). In our study, surgery was required in 6 patients after an episode of relapse with a recurrence rate of 33%, slightly higher than reported by other studies (11–28%) (1, 14). A recent survey of pediatric surgeons found that most respondents preferred chest tube insertion in the management of a first episode of PSP and performed surgery for a second episode, in the presence of bullae more than 2 cm in diameter, or for recurrent pneumothorax (25). Figure 1 shows the management flow-chart for PSP in our third level pediatric surgical ward.

Our study limitations include being retrospective and using a small cohort of patients. Prospective studies with a higher number of patients are encouraged to confirm our findings and establish evidence-based guidelines for the treatment of PSP in the pediatric population.

## Conclusion

According to our experience, we recommend that small PSP or clinically stable larger PSP be treated conservatively with observation alone. An X-ray is advised within 3 days of starting observation; if it shows a reduction of the PSP the patient could be discharged home. If the PSP has not decreased in size, considering a chest drain insertion is appropriate. Persistent air leak or episodes of relapse should be investigated with a CT scan and considered a strong indication to proceed with surgery.

## Data Availability Statement

The original contributions presented in the study are included in the article/Supplementary Materials, further inquiries can be directed to the corresponding author.

## Ethics Statement

Ethical review and approval was not required for the study on human participants in accordance with the local legislation and institutional requirements. Written informed consent from the participants' legal guardian/next of kin was not required to participate in this study in accordance with the national legislation and the institutional requirements.

## Author Contributions

Conceptualization: AB, EG, and JS. Methodology: GS and GC. Investigation: GS, GC, and DO. Data curation: GS, AB, MG, and DC. Formal analysis: GS and MG. Validation: AB, DC, EG, and JS. Writing—original draft preparation: GS, M-GS, MI, and DO. Writing—review and editing: GS, AB, DC, M-GS, and MI. Supervision: JS. All authors approved the final version of the manuscript and attest that they meet the ICMJE criteria for authorship.

## Funding

This work was supported by the Ministry of Health, Rome—Italy, in collaboration with the Institute for Maternal and Child Health IRCCS Burlo Garofolo, Trieste—Italy.

## Conflict of Interest

The authors declare that the research was conducted in the absence of any commercial or financial relationships that could be construed as a potential conflict of interest.

## Publisher's Note

All claims expressed in this article are solely those of the authors and do not necessarily represent those of their affiliated organizations, or those of the publisher, the editors and the reviewers. Any product that may be evaluated in this article, or claim that may be made by its manufacturer, is not guaranteed or endorsed by the publisher.

## Abbreviations

PSP: primary spontaneous pneumothorax
CT: computed tomography
LOS: length of stay
VATS: video-assisted thoracoscopic surgery.
