# Supplementary material for: Management of Primary Spontaneous Pneumothorax in a Third-Level Pediatric Surgical Center: A Retrospective Study
Source: Front Pediatr. 2022 Jun 27;10:945641. doi: 10.3389/fped.2022.945641 (PMC9271819; doi:10.3389/fped.2022.945641)
Supplement: Supplementary file 1 [file Data_Sheet_1.doc]

#	Sesso	Età	Altezza	Sede PNX	Peso	Dimensior	Degenza	TC sì/no	Blebs/boll	Interventc	Recidiva	intevento	Recidiva d
1	M	16		sx		4 cm	2 gg	sì	Sì	nulla	Sì, a sinistra	Chir	NO
2	M	16	184 cm	sx	72 kg	7 cm	6 gg	sì	No	Drenaggio	/	/	/
3	M	17		sx	64 kg	2,5 cm	4 gg	no	/	Niente	/	/	/
4	M	16		sx	66.5 kg	2,5 cm	10 gg	sì	sì	VATS + pleur	Sì	Chir	no
5	F	16	180 cm	sx	60 kg	4 cm	5 gg	Sì	no	Drenaggio	/	/	/
6	M	13		bilaterale	56 kg	0,7 cm	2 gg	no	/	niente	/	/	/
7	M	12		sx		1,4 cm	2 gg	no	/	niente	/	/	/
8	M	16		sx	65 kg	8 cm	3 gg	no	/	niente	/	/	/
9	M	16		sx	70 kg	4 cm	4 gg	sì	no	Drenaggio	dx	niente	/
10	M	16	185 cm	sx	60 kg	3 cm	9 gg	sì	sì	Drenaggio	dx + sx	Chir	SI
11	M	9	148 cm	sx	38 kg	5 cm	4 gg	sì	sì	drenaggio	3 volte a sx	Chir	SI
12	M	17		sx		11 cm	8 gg	no	/	drenaggio e v	/	/	/
13	M	15	172 cm	sx	44,5 kg	2 cm	2 gg	si	sì	niente	si, 2 a sx e 2	Chir	no
14	M	17	182 cm	sx	65 kg	4 cm	4 gg	no	/	niente	/	/	/
15	M	17		sx	62 kg	5,5 cm	7 gg	no	/	drenaggio	/	/	/
16	M	13	175 cm	sx	60 kg	1 cm	3 gg	no	/	niente	/	/	/
17	M	17		sx	50 kg	2,3 cm	17 gg	si	sì	drenaggio	si, a sinistra	Chir	NO
18	M	17	187 cm	sx	76 kg	4 cm	4 gg	no	/	drenaggio	/	/	/
19	M	14		sx	45 kg	2 cm	2 gg	no	/	niente	/	/	/
20	M	16	181 cm	sx	64,3 kg	4,5 cm	3 gg	no	/	drenaggio	/	/	/
21	M	17		sx		3,8 cm	2 gg	no	/	niente	/	/	/
22	M	17	190 cm	dx	76,5 kg	4 cm	3 gg	no	/	drenaggio	/	/	/
23	M	17		sx	85 kg	5 cm	1 gg	no	/	niente	/	/	/
